# Visual Sensitivity of Deepwater Fishes in Lake Superior

**DOI:** 10.1371/journal.pone.0116173

**Published:** 2015-02-03

**Authors:** Kelly A. Harrington, Thomas R. Hrabik, Allen F. Mensinger

**Affiliations:** Biology Department, University of Minnesota Duluth, 1035 Kirby Drive, Duluth, Minnesota, United States of America; University of Sussex, UNITED KINGDOM

## Abstract

The predator-prey interactions in the offshore food web of Lake Superior have been well documented, but the sensory systems mediating these interactions remain unknown. The deepwater sculpin, (*Myoxocephalus thompsoni*), siscowet (*Salvelinus namaycush siscowet*), and kiyi (*Coregonus kiyi*) inhabit low light level environments. To investigate the potential role of vision in predator-prey interactions, electroretinography was used to determine visual sensitivity for each species. Spectral sensitivity curves revealed peak sensitivity at 525 nm for each species which closely corresponds to the prevalent downwelling light spectrum at depth. To determine if sufficient light was available to mediate predator-prey interactions, visual sensitivity was correlated with the intensity of downwelling light in Lake Superior to construct visual depth profiles for each species. Sufficient daytime irradiance exists for visual interactions to approximately 325 m for siscowet and kiyi and 355 m for the deepwater sculpin during summer months. Under full moon conditions, sufficient irradiance exists to elicit ERG response to light available at approximately 30 m for the siscowet and kiyi and 45 m for the deepwater sculpin. Visual interactions are therefore possible at the depths and times when these organisms overlap in the water column indicating that vision may play a far greater role at depth in deep freshwater lakes than had been previously documented.

## Introduction

**Figure 1:**
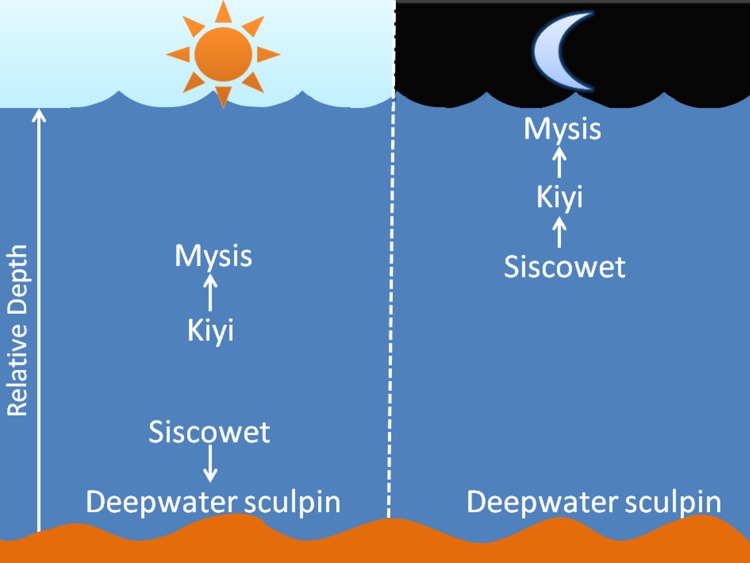
The main food web of the offshore waters of Lake Superior. The relative depth is plotted on the y axis with fish distribution shown both day (left) and night (right). Arrows indicate predation on particular species.

Lake Superior is the largest of the Laurentian Great Lakes and home to 38 fish species, including 19 nonnative species [1], with the majority of these fishes inhabiting the shallow, nearshore waters or surrounding watersheds. The cold deep, oligotrophic offshore waters of Lake Superior are relatively depauperate with fish density less than 6.9 kg/ha [2]. Although many invasive aquatic species have disrupted and/or become integrated into shallow water community, the deep waters of Lake Superior remain dominated by native species [3]. Piscivores such as burbot (*Lota lota*) and siscowet lake trout (*Salvelinus namaycush siscowet*) dominate the highest trophic levels and feed predominately on deepwater sculpin (*Moxocephalus thompsonii*) and/or kiyi (*Coregonus kiyi*) [4]. The deepwater sculpin and kiyi, along with the cisco (*Coregonus artedi*) form the second trophic level, and prey on a wide variety of zooplankton such as mysis (*Mysis diluviana*), scuds (*Diporeia* spp.), cladocerans, and copepods [3,5]. Thus, energy transfer in the deep, oligotrophic water of Lake Superior is mediated through a relatively simple food web (Fig. 1).

The diel vertical migrating (DVM) zooplankton, *Mysis diluviana*, is the primary conduit for energy flow from benthic waters to the surface, as it feeds diurnally on benthic detritus and switches to midwater phytoplankton and zooplankton during its nightly ascent [6,7]. Two planktivorous fish, deepwater sculpin and kiyi, prey primarily on the mysis, with the deepwater sculpin also consuming benthic amphipods (*Diporea* spp.) [3,8]. The siscowet is the most abundant piscivore in the lake [2,9] and its feeding habits are dictated by diurnal vertical migrations of the planktivores [7]. During the day, the siscowet remain in deepwater (>140 m) and prey primarily on benthic sculpin, while at night, they vertically migrate to consume kiyi which are following the migration of mysis [2,3,4,10,11].

Despite detailed information on the food web and diet of dominant species, little is known about the role that vision plays in mediating deepwater predator-prey interactions. While olfactory and auditory cues may be used for long range detection of prey, short range interactions usually are mediated by the mechanosensory lateral line or visual input [12]. Vision is often the main sensory modality in shallow, sunlit waters while the lateral line may be dominant in turbid and/or low light environments. To understand the role of vision, it is important to determine the visual and spectral sensitivity of the organism, and the intensity and spectral composition of downwelling irradiance. Predator-prey or population models often contain little sensory information and by incorporating sensory physiology, future models will better predict population structure and dynamics.

The fishes that comprise the deep water food web spend the majority of their time in a low light level environment. Following a pelagic larval stage, deepwater sculpin transition to the benthos and remain at depths ranging from 15 to 407 m [13,14,15,16] with the majority of the Lake Superior population inhabiting depths below 70 m. In contrast, siscowet and kiyi are midwater water fish that undergo diel vertical migration (DVM) with siscowet depth distributions ranging from the surface (night) to 407 m (day), while kiyi are found between 25 m (night) and 325 m (day) [7,10,17]. Therefore all three species spend the majority of their life in light limited environments.

The visual pigment sensitivity hypothesis [18] suggests that fish visual sensitivity corresponds with its light environment due to the adaptation of visual pigments. The spectral sensitivities of numerous marine species support this hypothesis [19,20,21,22,23,24,25,26]; marine organisms exhibit peak sensitivity to blue light as these are the predominate wavelengths at depth due to the filtering properties of seawater [27]. Marine fish contain the rhodopsin visual pigment, based on vitamin A_1_, which is well adapted for the detection of blue wavelengths. However, freshwater systems favor the transmittance of green light due to the high concentration of chlorophyll and other particulate matter in the water column [27,28]. The visual pigment porphyropsin, based on vitamin A_2_, is present in freshwater fish with its absorption spectrum matched to the predominant green downwelling light [29]. Freshwater fish utilize porphyropsin, or in conjunction with rhodopsin for detection [21,30].

Historically, deep sea fishes received more attention for their visual ability at depth than freshwater fish, creating a gap in the knowledge of the visual characteristics among deep water marine and freshwater fishes [28]. The clear, offshore waters of Lake Superior allow greater light penetration compared to other freshwater systems, and offer an opportunity to examine the visual sensitivity of deep water fishes in a freshwater system. The goal of the current study was to characterize previously unmeasured visual sensitivity of deep water fishes in Lake Superior and to determine the potential role of vision in mediating predator-prey interactions. Electroretinography was performed on three species of deep water fish found in Lake Superior to determine dark adapted spectral sensitivity and to compare each visual system to the prevailing light environment. The fishes’ visual sensitivity was combined with estimates of the transmission of light in Lake Superior to model the depths at which vision may mediate predator-prey interactions.

## Materials and Methods

### Fish Collection

Siscowet, deepwater sculpin, and kiyi were collected via daytime bottom trawls in the Apostle Islands region of Lake Superior, east of Stockton Island (Lat: 6° 54.751 Long: 90° 30.611) on November 13, 2012 and June 26, 2013 with the permission of the Wisconsin Department of Natural Resources. No invasive species were collected during the trawl and no endangered species were harmed during the collection procedure. Fish were collected at depths ranging from 100 to 117 m during 10 minute bottom trawls using a 12 m Yankee bottom trawl. Immediately after removal from the net, fish were submerged in a solution of lake water consisting of 0.0024% tricaine methanesulfonate (MS-222, Sigma Chemical Co., St. Louis, MO), 0.026% Stresscoat (Mars Fishcare North America Inc., Chalfont, PA), and 0.5% Instant Ocean (Aquarium Systems Inc., Mentor, OH) in 570 L plastic holding tanks in 6°C water. After 2 minutes, kiyi and siscowet swim bladders were deflated using 14 gauge veterinary needles (QC Supply, Schuyler, NE), the incisions treated with betadine (Purdue Products L.P., Stamford, CT), and the fishes placed back in the holding tanks. After an additional five minutes, fish were transferred to two 285 L transportation tanks at 6°C containing lake water solutions of 0.0002% MS-222, 0.026% Stresscoat, and 0.5% Instant Ocean. These tanks were then transported to the University of Minnesota Duluth. Throughout the entire capture and transport process, the water was aerated with compressed O_2_ via 5” Deluxe Bubble Disks (Penn Plax, Hauppauge, NY).

At the University of Minnesota Duluth, the sculpin, kiyi, and siscowet were placed into 40 L, 575 L, and 1900 L aquaria, respectively, equipped with mechanical, chemical and biological filtration using Penn-Plax Cascade 1500 canister filters. Prior to arrival, all tanks were aerated with compressed O_2_ for three days. Instant Ocean was added to all tanks to achieve 0.5% salt concentration. Carbon filtration was used during oxygen treatment, but was removed upon Stresscoat treatment. Tanks were treated with 0.026% Stresscoat one day prior to fish arrival, and were aerated with pure oxygen for four days after arrival, and carbon filtration resumed seven days post trawl. Water temperatures were maintained between 3 and 6°C. All tanks were maintained in refrigerated dark rooms and were illuminated indirectly by dim red light (Sunbeam 40 W red light bulb) when necessary for observation, fish selection, and tank maintenance. Water quality (pH, temperature, ammonia, nitrate, nitrite, and oxygen concentration) was monitored twice daily for the first 2 weeks, daily for weeks 3 and 4, and twice weekly thereafter. Feeding was initiated 48 hours after arrival and fishes were provided frozen mysis, with kiyi and siscowet supplemented with live mysis and shiner minnows when available. Food was provided every other day and uneaten food was removed from tanks within 24 hrs.

### Electroretinogram Preparation

All experimental procedures were conducted in a dark room illuminated by dim red light (15 W light bulbs with Kodak GBX-2 dark red safelight filter). All species underwent the same protocol and each fish was anesthetized with buffered (4.5% sodium phosphate dibasic, 1.1% potassium phosphate monobasic in d_i_H_2_O, Sigma Chemical Co., St. Louis, MO) 0.002% MS-222 with pH maintained between 7.0 and 7.4. A tail pinch was used to confirm that the surgical plane for anesthesia was achieved [31]. The fish was then immobilized by an intramuscular injection of pancuronium bromide (0.001 to 0.100%; 0.0004–0.0030% of body weight) dissolved in 0.9% NaCl. The fish was placed on a moist sponge in a 45 × 11 × 9 cm experimental tank and submerged up to the ventral border of the eyes. The experimental tank was housed within an opaque metal Faraday cage (77 × 67 × 96 cm) to eliminate instrumentation light from interfering with dark adaptation. Buffered 0.002% MS-222, maintained at 4°C (420 W Teco SeaChill Aquarium Chiller, Teco model SCTR20, Ravenna, Italy), was circulated continuously over the gills through an intraoral tube to maintain the surgical plane of anesthesia throughout the experiments. Upon completion of testing, organisms were either revived with fresh water (0.5% salt concentration) or sacrificed by immersion in 0.5% MS-222 for 1 hr.

This study was carried out in strict accordance with the recommendations in the Guide for the Care and Use of Laboratory Animals of the National Institutes of Health. The protocol was approved by the Institutional Animal Care and Use Committee of the University of Minnesota (Protocol: 1205A13881). All surgery was performed under MS-222 anesthesia, and all efforts were made to minimize suffering.

### Electroretinogram Collection

Light stimulus was provided by a 100 W quartz tungsten-halogen lamp (Newport model 6333, Stratford, CT) powered by a constant current power supply (Newport model 68938). The stimulus duration was regulated by an Oriel Electronic Shutter (model 76994) and Controller (model 76995) that delivered a square wave light pulse with 3.0 ms delay, 3.0 ms rise time, and 5.0 ms fall time. The light was passed through a monochromator (Newport model 77250). Neutral density filters (0.1 to 3.0) were used to regulate light intensity. A fiber optic light pipe (Newport model 77632) was used to transmit the light to the eye. Light intensity was measured using a radiant power energy meter (Ophir model 70260) and probe (Ophir model 70268). A 0.20 mm diameter silver-silver chloride recording electrode was inserted into the vitreous of the eye through an incision at the limbus, and a reference electrode was placed in the center of the frontal bone between the eyes. ERGs were amplified using World Precision Instrument, Inc. amplifier (1000×, 1 Hz low pass, 3 kHz high pass, model DAM50; Sarasota, FL), filtered using a 60 Hz notch filter, recorded with PowerLab 4SP (AD Instruments, Castle Hill, Australia), and stored using Lab Chart7 (AD Instruments, Castle Hill, Australia) software on a portable computer.

All fishes were dark adapted for 30 minutes prior to testing. A 200 ms flash of monochromatic light of different wavelengths was used to elicit the ERG. Wavelengths from 400 to 700 nm at 25 nm intervals were used as the stimulus with the presentation order randomly determined. Stimulus intervals were determined for each species by presenting consecutive flashes to control fish to determine the delay required to produce the same response amplitude to minimize photobleaching. Interflash intervals ranged from 30 s for the kiyi to 190 s for the deepwater sculpin. Experiments were attempted within the same time period each day to minimize intraspecfic circadian differences. Deepwater sculpin and siscowet experiments were initiated between 1030 and 1530. However, due to the compromised physiology of the kiyi, experiments were initiated as soon as fish were transported to the lab which resulted in trials being conducted between 0025 and 1030 within the first week following the trawl.

The b-wave amplitude (baseline to peak) of the ERG was used as the response criterion. The minimal b-wave amplitudes were consistently encountered in response to short wavelength light (≤ 425 nm) and therefore the b-wave amplitude at 400 nm was set as the minimal criterion response for each fish. Although b-wave amplitudes ranged up to 71 mV, the amplitudes were often less at the shorter wavelengths. Therefore the b-wave amplitude of 5 μV was set as the criterion response at 400 nm. This amplitude insured that at least a 5 μV response was attainable throughout the wavelengths tested. Wavelengths were reduced in intensity by neutral density filters until the b-wave amplitude equaled 5 μV for each wavelength tested. The corresponding irradiance to achieve the criterion response at each wavelength was used to generate spectral sensitivity curves for each species.

### Light Attenuation Calculation

Existing measurements of downwelling light for Lake Superior are limited to relatively shallow depths. Therefore, downwelling irradiance was calculated from sources of surface irradiance and light attenuation coefficients for Lake Superior. Total surface irradiance on Lake Superior in August ranged from 122 (afternoon) to 1832 (noon) μ einsteins m^−2^/sec^−1^ for photosynthetically active radiation (PAR) which measures the total energy for the visible light spectrum of 400–700 nm [32]. Photoreceptors are photon detectors and not energy detectors, and therefore it was necessary to convert the irradiance to photons^−1^ cm^−2^. The approximate middle of the surface irradiance range, 1000 μ einsteins m^−2^/sec^−1^ was arbitrarily chosen as the daytime light intensity. The following equation was used to convert this value to photons s^−1^ m^−2^:

$$E=E_{q}*{PAR}_{\lambda }*A*B$$ Eq. 1

E = energy in photon s^−1^ m^−2^

E_q_ = Energy in quanta units [32]

PAR_λ_ = percentage of incident solar radiation or moonlight for each wavelength [33]

A = 0.22; conversion of quanta to W m^−2^ [34]

B = 5.05 x 1015 * λ; conversion of W m^-2^ to photon s^−1^ m^−2^ at each λ (nm) [35]

Most available moonlight values for Lake Superior were reported in lux which corresponds to human visual sensitivity and was not accurate to use for fish vision. It was not possible to convert lux to irradiance without knowing the spectral sensitivity of the detector. Therefore, moonlight intensities were used from Cramer *et al.* [36] who reported wavelength specific surface irradiance for moonlight in Arizona (peak value of 2.67 μW m^−2^ at 600 nm). The values were converted to W m^−2^ and then transformed to photon s^−1^ m^−2^ using B in equation 1.

To determine the depth at which sufficient irradiance was available to evoke the criterion b-wave response, the Beer-Lambert law as used to calculate light attenuation at depth. The surface intensity was calculated for each wavelength using equation 1 and the Beer-Lambert law was used to calculate intensity available at depth:

$$E_{\left(z,\lambda \right)}=E_{\left(0,\lambda \right)}e^{-[k_{\left(\lambda \right)}z]}$$ Eq. 2

E_(z,λ)_ = spectral irradiance at depth z (m) and wavelength λ (nm)

E_(0,λ)_ = incident surface spectral irradiance

k = spectral irradiance attenuation coefficients [33]

Seasonal changes in water clarity result in different k_PAR_ values for Lake Superior with clearer water present in the spring and summer (k_PAR_ = 0.1), and fall characterized by a reduction in water clarity (k_PAR_ = 0.3). Therefore different spectral irradiance attenuation coefficients were used to determine light attenuation under different water conditions [33]. Data for winter months were not available; however ice and snow cover, low sun angles, and shorter days can limit surface irradiance [37] making winter the most probable time for minimal light availability at depth.

## Results

All species survived trawling and transport to the University of Minnesota Duluth. Despite remediation of gas bladder expansion in the kiyi, fish continued to exhibit buoyancy problems and survived less than a week in captivity. Therefore, all kiyi were tested within 4 days of capture. Although siscowet displayed minor buoyancy problems, these quickly disappeared within one week of capture and both the siscowet and sculpin have been maintained over a year in captivity indicating no long term physiological effects of trawling. Siscowet were tested within 3 weeks of capture and sculpin within 2 months of capture.

### Spectral sensitivity

**Figure 2:**
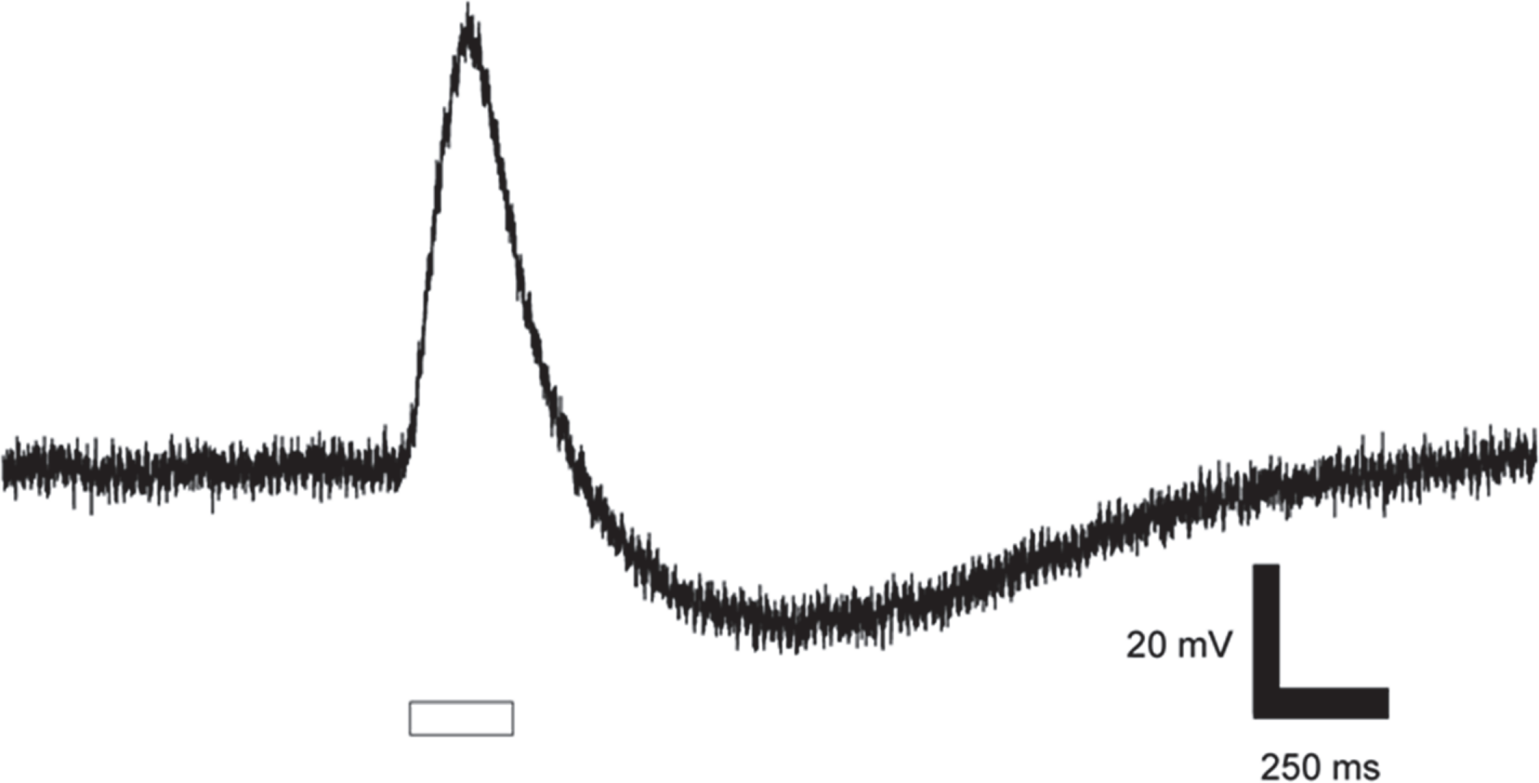
An electroretinogram with amplitude of the b-wave (mV) plotted vs time. The ERG was recorded from kiyi in response to 550 nm light. The open rectangle indicates the onset and offset of the 200 ms flash.

Deepwater sculpin were the most susceptible to short interflash intervals and needed a minimum of 190 seconds between flashes to avoid transient “bleaching”. Lake trout required 80 seconds, while 30 second interflash intervals were sufficient for kiyi to recover full sensitivity. The dark adapted retinas displayed a strong b-wave and no evidence of an a-wave (Fig. 2).

**Figure 3:**
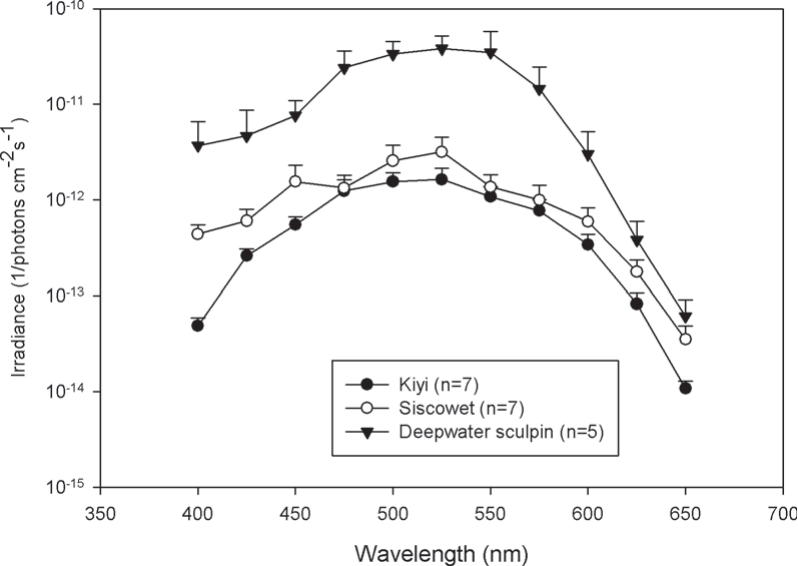
The average irradiance (1/photons cm^−2^s^−1^) needed to invoke the criterion response is plotted versus wavelength (nm) for kiyi (black circles), siscowet (open circles), and deepwater sculpin (triangles). Lines connecting the symbols are for illustrative purposes only. Error bars = 1 SE.

Visual spectral sensitivity curves for dark adapted siscowet, kiyi, and deepwater sculpin were constructed using ERG responses to monochromatic light of different wavelengths. All three fishes exhibited maximum sensitivity at 525 nm with relatively broad sensitivity from 500 to 550 nm and markedly decreasing sensitivity to wavelengths < 475 nm and > 575 nm (Fig. 3).

### Visual depth profiles

**Figure 4:**
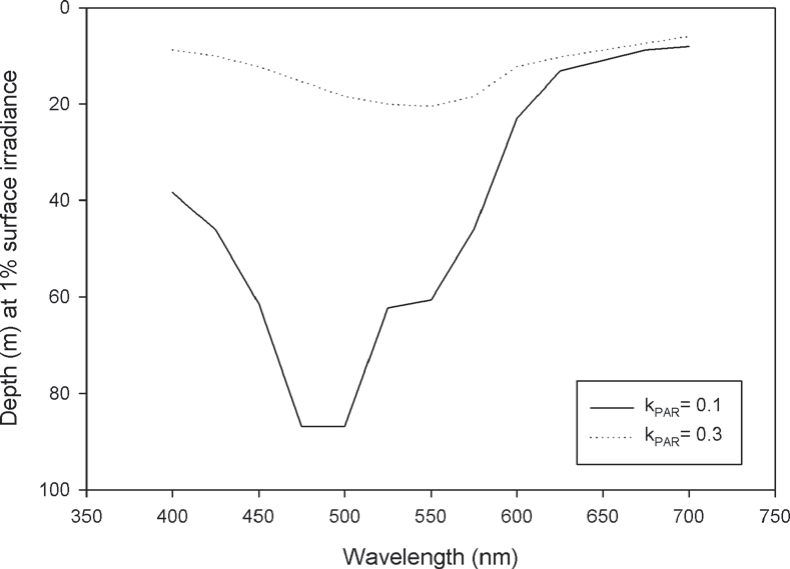
The depth at which 1% of surface irradiance occurs under spring summer (k_PAR_ = 0.1) conditions (solid line) and fall (k_PAR_ = 0.3) conditions in Lake Superior.

To illustrate the differences in light attenuation for each wavelength and season (spring/summer vs fall) the maximum depth at which solar irradiance was reduced to one percent of surface values in Lake Superior was plotted (Fig. 4). 475 to 500 nm light was maximally transmitted in the spring/summer, while fall shifted the spectrum to slightly longer wavelengths with 550 nm light showing maximum depth penetration.

**Figure 5:**
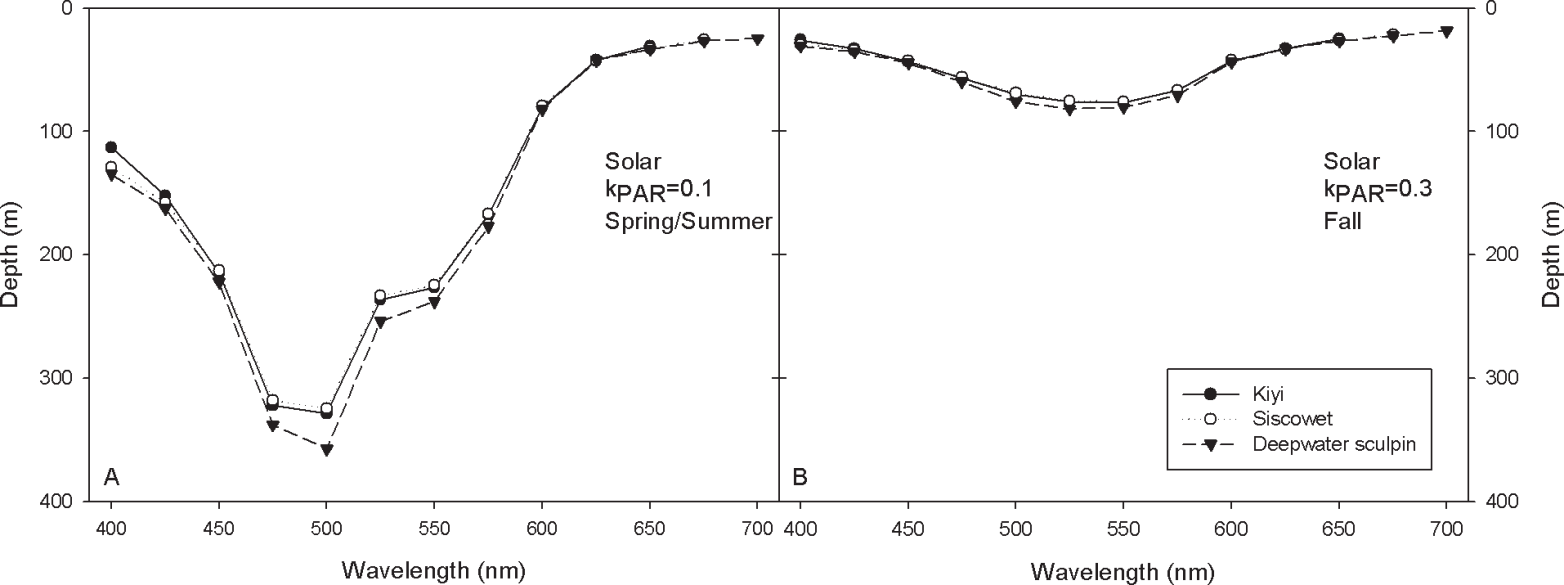
The maximal depth at which sufficient downwelling irradiance is available to elicit an ERG under full sun conditions is plotted versus depth (m) for kiyi (black circles), siscowet (white circles), and deepwater sculpin (black triangles) for (A) spring/summer (k_PAR_ = 0.1) and (B) fall (k_PAR_ = 0.3) conditions in Lake Superior.

Visual depth profiles were created to determine the maximum depth at which sufficient irradiance is available to elicit the criterion ERG amplitude in the three species of fish tested to approximate the depth at which fish can detect light. All three species had sufficient visual sensitivity to detect 500 nm light at depths greater than 325 m during the day in spring/summer (k_PAR_ = 0.1) months (Fig. 5A). Longer wavelengths (≥ 625 nm) were rapidly attenuated and not detectable deeper than 40 m. Deepwater sculpin displayed broader sensitivity between 475 to 550 nm than the other two species, but outside of these wavelengths, all fishes had similar spectral sensitivity curves.

Visual depth profiles changed with decreased water clarity (k_PAR_ = 0.3) with downwelling daytime irradiance attenuating rapidly. Intensity was sufficient to elicit ERGs at maximum depths of approximately 75 m for all species (Fig. 5B). Again, all species demonstrated similar profiles with maximum sensitivity between 500 and 525 nm, with the deepwater sculpin retaining a slight advantage in detection of wavelengths between 500 and 575 nm.

**Figure 6:**
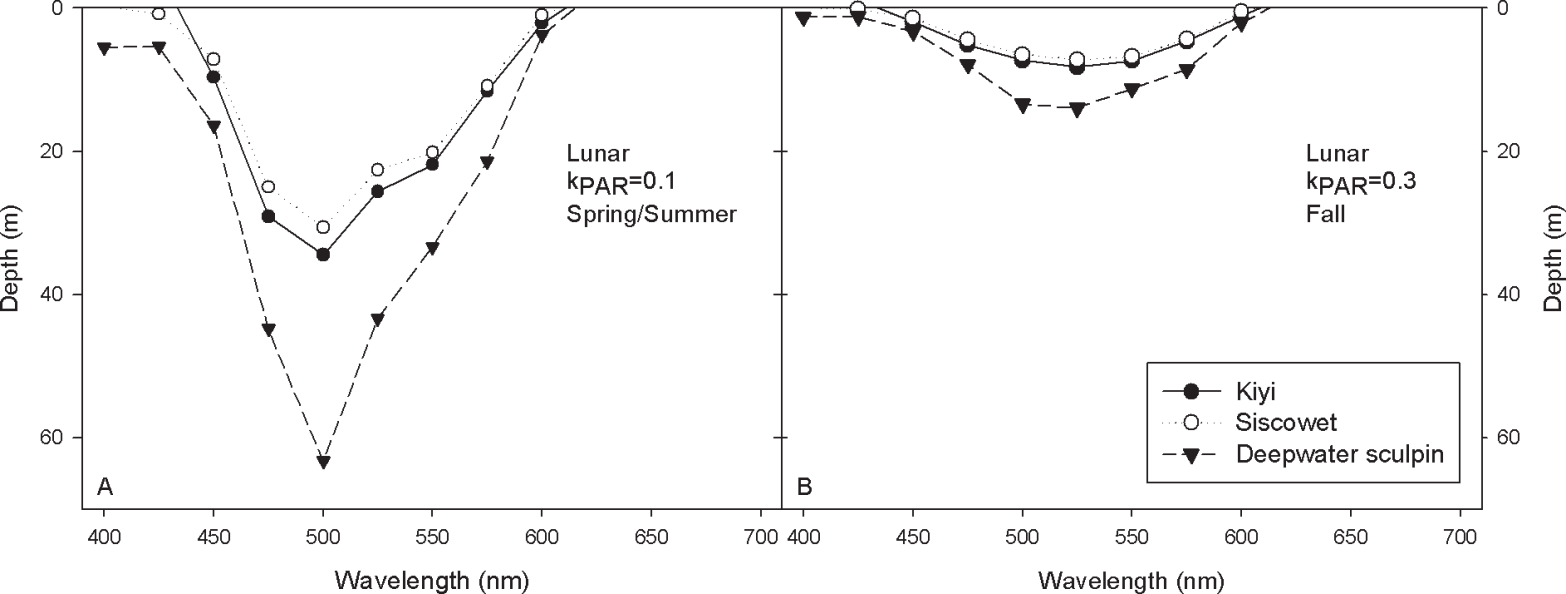
The maximum depth at which sufficient downwelling irradiance is available to elicit an ERG under full moon conditions is plotted versus depth (m) for kiyi (black circles), siscowet (white circles), and deepwater sculpin (black triangles) for (A) spring/summer (k_PAR_ = 0.1) and (B) fall (k_PAR_ = 0.3) conditions in Lake Superior.

During the spring and summer months, kiyi and siscowet nocturnal visual sensitivity was sufficient to detect 500 nm light, under full moon conditions to depths of approximately 30 m, while deepwater sculpin could detect downwelling irradiance to 63 m (Fig. 6A). During the fall, maximal moonlight penetration was less than 15 meters for sufficient intensity to elicit ERGS, with depths estimated at 8 m for kiyi and siscowet and 14 m for the deepwater sculpin for 525 nm light (Fig. 6B). Sensitivity at either end of the visual spectrum was greatly reduced under both diurnal and nocturnal conditions for all organisms.

## Discussion

The deep water fishes of Lake Superior exhibited similar spectral sensitivities with peak sensitivity at 525 nm that correlated to the predominant downwelling wavelengths. Based on visual sensitivity and light attenuation estimates in Lake Superior, sufficient irradiance exists to mediate visual interactions throughout most, if not all, of the species daytime depth distributions. Additionally, sufficient downwelling irradiance is available under certain nighttime conditions to provide sufficient intensities to elicit ERG responses in the DVM species during nocturnal ascents.

Light exposure on the surface can transiently or permanently damage the retinas of mid water animals [38,39] although its effect on fish remains to be determined. The fishes in our study were trawled from 100 to 115 m during both day and night and were exposed to sunlight or deck lights during capture and transport, and the effects of light exposure on the visual sensitivities of these Lake Superior fishes are uncertain. However, every effort was made to maintain fish under dim red light conditions following capture, and during ERG testing. As all animals displayed a robust ERG when stimulated, and displayed spectral sensitivity curves consistent with other freshwater species, it appears that most, if not all retinal components were intact. Control sculpins and siscowet were maintained for months in captivity without detectable changes in visual or spectral sensitivity suggesting that any light damage that may have occurred to the retina probably was minimal or transient. Kiyi proved less robust and buoyancy issues due to trawling resulted in short survival times (generally less than one week). As such, kiyi were maintained for a shorter recovery period under dim light before testing. All kiyi exhibited a strong response to monochromatic light indicating retinal function was maintained, however, given their compromised physiology, their results should be treated with a degree of caution.

Due to both specialized morphological retinal adaptation and the optical clarity of open ocean water, it has been estimated that mid water fish can detect downwelling light to 1000 m [40,41]. However, most lakes contain more particulate matter, such as non-algal particulates and colored dissolved organic matter [42,43] that increase light attenuation and shift the downwelling spectral irradiance to longer wavelengths than in salt water. Specialized retinal adaptations to increase visual sensitivity, such as a multi-layer retina, have not been identified in freshwater fish. Estimates of fish visual sensitivity at depths greater than 100 m are rare for freshwater fishes. The deep, oligotrophic Lake Superior provided an excellent venue to examine fish visual capabilities in deep freshwater systems.

Many papers on fish vision report surface or underwater light intensities in photometrics (lux) which are based on human visual perception and not accurate for most animal species [35]. Therefore irradiance values on Lake Superior were obtained from Fahnensteil *et al.* [32]. However, the spectral distribution of light is not constant from 400 to 700 nm and therefore the percentage of energy for each wavelength was obtained for Jerome *et al.* [33] as well as the extinction coefficients for water. As nocturnal irradiance for Lake Superior was not available outside of photometric units, moonlight irradiance recorded in southern Arizona was substituted as the study provided a detailed irradiance for each wavelength tested [36].

The ERG has long been used to assess spectral sensitivity by determining the electrical potential of the retina. In light adapted retinas, ERG waveforms include an a-wave generated by the photoreceptor hyperpolarization upon initiation of a light stimulus, and a b-wave, originating from Müller cell and bipolar cell depolarization [44]. When dark adapted, the a-wave is absent and allows a more precise determination of the b-wave amplitude. As the Lake Superior fish inhabit minimal light environments, the dark adapted retina was more consistent with environmental conditions and was used to assess spectral sensitivity. Therefore given the low light intensities used for stimulation and the dark adapted condition of the retinas, the information presented is limited to scotopic visual sensitivity most likely mediated by the rod photoreceptors.

The ERG provides a mechanism for minimally invasive sampling and allows the fish to be used also in behavioral studies. While it is an effective tool to measure spectral sensitivity, it does not assess the central visual pathways and brain centers involved in image formation, and therefore cannot be directly correlated with image formation. However, for the b-wave to be induced, sufficient light must be absorbed by the photoreceptors to stimulate the bipolar and Müller cells to allow the electrical potential of the retina to be detected, strongly suggesting that fish can centrally process this light. Additionally, the current path to the differential extracellular electrodes, one in the vitreous and one on the epidermis, necessitates that the electrical signal must travel through different tissue and unlike intracellular electrodes, some current will be lost before detection. Therefore, it is highly probable that visual sensitivity may be greater than values reported. However, given the challenge of capturing and maintaining these deep water species, the ERG provides the best proxy to assess visual sensitivity in these fishes. Therefore, for the purposes of this paper, visual sensitivity is defined as the minimal irradiance sufficient to elicit the criterion b-wave amplitude in a dark adapted retina.

The spectral sensitivity curves showed all three species had broad spectral sensitivities that correlate with prevailing downwelling light in Lake Superior. The spring and summer water column is clearer and contains less particulate matter than fall, thus allowing greater light transmission to depth with 500 nm wavelengths penetrating the furthest. In the fall, the greater suspension of particulate matter increases light absorbance and shifts the deepest penetrating light to 550 nm. Thus, the peak spectral sensitivity of 525 nm is well adapted for the seasonal differences in downwelling light. The reduced spectral sensitivity to the longer wavelengths is consistent with their deep water environment because red light is quickly attenuated in Lake Superior [25,27]. Similarly, shorter wavelengths are absorbed relatively close to the water’s surface in freshwater systems, although attenuation of 400 to 450 nm light occurs more slowly than red wavelengths [18,25,27]. Thus the visual pigments in the fish are adapted to seasonal changes in the prevailing downwelling spectrum; the broad sensitivity range exhibited between 500 and 550 nm is consistent with Clarke’s sensitivity hypothesis that visual pigments are matched to downwelling light.

These findings offer a unique opportunity to explore possible visually mediated behavior in a deep lake system by correlating spectral sensitivities to the estimated irradiance at depth. Many studies of fish vision have investigated spectral sensitivity using electroretinography [45] or microspectrophotometry to understand the maximum wavelength of perception for retinal components [19,23,46,47,48,49], however, few have compared the visual sensitivities of fishes to a detailed profile of the light available in the natural environment [50,51,52].

Deep water marine fishes are often physiologically compromised both by the physical impacts of the trawl net and/or pressure differentials encountered during retrieval. Due to the challenges of capturing marine fish in good condition and maintaining these often moribund fish long enough to perform electrophysiology, the spectral sensitivity and visual depth profiles have often been extrapolated from retinal histology or microspectrophotometry. While these techniques have provided a wealth of information on the evolutionary adaptation of fish visual systems to low light levels, they do not provide empirical information about visual sensitivity at depth.

Many estimates of fish vision at depth are based on indirect evidence or extrapolations of non physiological experiments. Clarke [18] extrapolated from psychophysical visual experiments the depths in which the freshwater sunfish could see in both freshwater and marine environment and suggested visual capabilities down to 430 m in the Sargasso Sea (an environment that the fish do not inhabit) and to approximately 110 m depth in Lake Superior. Nicol [53] suggests that retinal adaptation of midwater fish allow vision to 1000 m in the open ocean. However, physiologically determined minimum light responses have been rare due to previously mentioned issues. The ability to retrieve and maintain fish under low light level conditions for several months after capture allowed investigation of visual sensitivities of deep dwelling fresh water fish. Combined with surface irradiance and the attenuation coefficients of Lake Superior, it provided the opportunity to model visual sensitivity based on retinal responses. As the deep water food web is an important component of the Lake Superior ecosystem, it is imperative to understand the sensory physiology of the animals to produce accurate predator-prey models.

The deepwater water sculpin proved exceptionally sensitive to light stimuli with over 3 minutes required between flashes to obtain successive equal amplitude ERGs. Direct interspecific comparisons of maximum visual sensitivity are not possible using the ERG as eye size and electrode position can influence b-wave amplitudes between fishes, however, the long interflash interval needed to regain full ERG amplitudes indicates that the deepwater sculpin maybe the most sensitive of the three species. This is consistent with its deepwater benthic adult phase which places it in a low intensity light environment. The models show sufficient visual sensitivity to potentially mediate predator-prey interactions throughout the majority of its range. As the deepwater sculpin is the preferred prey of siscowet, its greater visual sensitivity may allow it to detect the siscowet at sufficient range to evade predation. Its benthic position provides an additional advantage as it can discern the predator silhouettes illuminated by the downwelling light while the siscowet faces the more difficult task of visualizing benthic prey against a dark background. Although at its average depth insufficient light is available for visual function at night, its main predator undergoes DVM and therefore vision may not be necessary during this time.

In contrast, the siscowet and kiyi visual sensitivities are sufficient to allow daytime vision throughout most of these fishes’ diurnal depths and full moonlight could provide sufficient irradiance to allow vision from 30 to 60 m depth. Depending on the intensity of the isolume that they follow, both species may have sufficient visual sensitivity to use vision to feed or avoid predation at night.

The three fish species that comprise the offshore food web of Lake Superior have evolved spectral sensitivity to match the prevailing downwelling light. Their visual sensitivity appears sufficient to utilize visual cues for predator avoidance and prey capture. While other sensory modalities may be important for long range detection, most short range predator-prey interactions are mediated by the mechanosensory lateral line and/or vision. Teleosts are certainly capable of finding prey in complete darkness, although, at best range, the mechanosensory lateral line range is estimated to be one or two body lengths with neurophysiological studies on free swimming fish feeding on natural prey suggesting even shorter distances of less than a body length [54]. Vision can greatly extend this range; however, optical conditions in the aquatic environment can be highly variable. Future studies implementing these spectral sensitivity findings can be incorporated into laboratory studies investigating fish reaction distance under diminishing light intensities. By simulating environmental conditions, foraging mechanisms for offshore fish can be further understood.
